# Routine preoperative CT: Ready to roll or a step too far?

**DOI:** 10.1111/jocs.16397

**Published:** 2022-03-10

**Authors:** Pradeep Narayan, Gianni D. Angelini

**Affiliations:** ^1^ Rabindranath Tagore International Institute of Cardiac Sciences Narayana Health Kolkata India; ^2^ Bristol Heart Institute Bristol University Bristol UK

**Keywords:** CABG, preoperative CT, stroke

The incidence of stroke after coronary artery bypass grafting (CABG) is around 1.3% in the Society of Thoracic Surgeons database but carries a mortality of almost 20%.^1^, ^2^ The stroke rate is even higher with aortic valve replacements with the Determining Neurologic Outcomes from Valve Operations (DeNOVO) study reporting stroke rates as high as 17%.^3^ It is also likely that variations in the definition of stroke lead to underreporting of this event. There is thus, no denying that stroke remains the Achilles heel of cardiac surgery and efforts must be made to mitigate its occurrence.

The case by Osorio–Jaramillo published in this issue of the journal reignites the discussion on the value of computerized tomographic  (CT) imaging as a routine preoperative investigation before cardiac surgery.^4^ The identification of a mass on the ascending aorta led to a revision in operative strategy whereby along with the initially planned CABG, concomitant supracoronary replacement of the ascending aorta was also performed. It is likely that in the absence of modification of surgical strategy this unexpected mass in the ascending aorta may have led to a perioperative stroke with serious adverse outcomes.

Atheromatous disease of the aorta not only increases the risk of perioperative stroke but also impacts upon long term mortality.^5^, ^6^, ^7^ The stroke rate has been shown to be quadrupled in presence of atheroma in the proximal aorta.^5^ Prevention of these adverse outcomes thus hinges on the identification of the atheromatous aorta. While this has been attempted intra‐operatively by manual palpation of the aorta the reliability with manual palpation is low with the additional risk of plaque embolization.^7^, ^8^ Trans‐esophageal‐echocardiography (TEE) has also been used to detect atherosclerotic aorta but has limited access to the ascending aorta and arch and the sensitivity is only marginally better than manual palpation.^9^ Epi‐aortic ultrasound is significantly better than both manual palpation and TEE and is recommended for identification of atheromatous plaques (Class IIa, level of evidence C)^10^ The downside of Epi‐aortic ultrasound is it is operator dependent and more importantly the information is available only after a sternotomy has been performed. Preoperative planning and a considered discussion with the patient is thus not possible. Multidetector computed tomography (MDCT) is being used increasingly to diagnose the atheromatous aorta and its superior spatial resolution coupled with 3D reconstruction capabilities makes it an attractive option in the preoperative setting.^11^

Preoperative CT has been used in the past predominantly in the re‐operative setting. Studies comparing patients undergoing reoperative surgery have reported a reduction in stroke rates in patients undergoing preoperative CT scans.^12^ Apart from stroke, there was also a reduction in sternotomy‐related injury, complication rates^13^, ^14^ and this resulted in improved perioperative outcomes.^15^ There are contradictory findings with regard to the effect of preoperative CT scans on mortality with some studies suggesting improved outcomes^13^, ^15^ while others suggest an increase of mortality in these patients.^12^ While the findings of all these studies may be influenced by several factors and are open to questioning one fact remains indisputable that most studies report a change in either surgical access, the strategy of cannulation, or even operability.^16^ While the indication for a preoperative CT in re‐operative surgery is essential to assess the anatomical orientation of structures and especially the grafts after CABG; its role in primary cardiac surgery is to evaluate the extent of atherosclerosis of the ascending aorta and arch.^16^

While the usefulness of preoperative CT scans is intuitive, robust data on improvement with this strategy is sparse, especially in routine or low‐risk patients. In high‐risk patients use of preoperative CT scans in patients undergoing cardiac surgery has been shown to be associated with a reduction in stroke rates as well as mortality.^17^ However, the groups compared were from two different time periods. Another study that carried out preoperative CT scans in patients undergoing CABG except those who required emergency surgery or had renal failure demonstrated a change of strategy in nearly half the patients.^18^ The change in strategy included avoidance of cannulation and cross‐clamping by using off‐pump anaortic techniques, alteration in conduit selection and grafting strategy, as well as replacement of ascending aorta and additional procedures on the carotid and renal arteries. Besides, preoperative CT identified malignancy in some patients as well.

The concerns with using preoperative CT scans include radiation exposure, contrast‐induced nephropathy, additional cost, and utilization of resources which may be an important consideration in resource‐depleted countries and state‐funded healthcare systems. Even though, ultra‐low‐dose CT scanning without contrast enhancement can satisfactorily identify an atheromatous aorta thus ameliorating some of the concerns the benefit of preoperative CT scan is still not established. Studies that have compared the role of preoperative CT in improving outcomes, especially in primary surgery are few and suffer from considerable heterogeneity.

While some of the studies have used contrast‐enhanced CT scans others use non‐contrast CT for reporting outcomes. Considerable variability also exists in the case‐mix with CABG, valve surgeries, and CABG with concomitant valve surgery being included in varying proportions in different studies all of which further muddy the water. Mortality has been defined at 30‐day in some and as in‐hospital in others making a comparison across studies extremely unreliable. The definition of stroke in these studies still needs to be standardized. The current definition of stroke as recommended by the new American Heart Association/American Stroke Association statement has modified the definition such that in addition to clinical features of stroke, evidence of cerebral emboli by diffusion‐weighted magnetic resonance imaging also constitutes stroke.^19^

Preoperative CT scans have shown potential and should not be dismissed, however, large scale randomized studies would be needed to confirm its place in routine preoperative evaluation of cardiac surgical procedures. The results of “Ultra low‐dose chest CT with iterative reconstructions as an alternative to conventional chest X‐ray before heart surgery (CRICKET study)” are awaited (NCT02173470). The study is a multi‐centric randomized controlled trial comparing preoperative chest X‐rays and those undergoing an additional preoperative noncontrast‐enhanced chest CT among patients undergoing cardiac surgery.^20^ The outcomes studied include stroke rate and alteration of surgical strategy. Until the time more conclusive evidence is available, routine preoperative CT scans seem a step too far and can perhaps only be justified in re‐operative surgery and in patients at high risk for developing a peri‐operative neurological event.

## CONFLICTS OF INTEREST

The authors declare no conflicts of interest.
